# Placement of p6pol between tandem repeat HIV-1 protease domains reduces Gag cleavage efficiency

**DOI:** 10.1186/1742-4690-10-S1-P98

**Published:** 2013-09-19

**Authors:** Tin-An Chou, Kuo-Jung Huang, Chin-Tien Wang

**Affiliations:** 1 Institute of Clinical Medicine, National Yang-Ming University School of Medicine, Taipei, Taiwan; 2 Department of Medical Research and Education, Taipei Veterans General Hospital, Taipei, Taiwan

## Background

HIV-1 protease (PR) is encoded by *pol*, which is initially translated as a Pr160*^gag-pol^* polyprotein by a ribosomal frameshift event [1]. Pr160*^gag-pol^* is incorporated into virions via interactions with assembling Pr55*^gag^*. The PR-mediated proteolytic cleavage of Pr55*^gag^* and Pr160*^gag-pol^*, known as virus maturation, is essential for the acquisition of viral infectivity. Within the Gag-Pol, the p6gag is truncated and is replaced by a transframe domain referred to as p6* or p6pol. Removal of p6pol improves Gag-Pol autoprocessing, suggesting that p6pol is involved in regulation of PR activation [2]. However, overlapping of p6gag/p6pol reading frame hampers generic approach to studying p6pol biological function. To assess the p6pol contribution to PR-mediated virus maturation without affecting p6gag reading frame, we introduced an extra copy of p6pol-PR or PR coding sequence at the PR C-terminus.

## Materials and methods

PCR-amplified p6pol-PR or PR fragments were inserted at the PR C-terminus of an *env*-deleted HIV-1 proviral vector. Each of the constructs was transiently expressed in 293T cells, and virus assembly and processing were analyzed by Western blot. Virus infectivity was determined by a single-cycle infection assay.

## Results

HIV-1 mutants containing tandem repeat PR domains were severely defective in virus particle production due to enhanced Gag cleavage. Inactivation of the proximal PR affects Gag cleavage efficiency at a greater extent than inactivation of the distal PR. Placement of p6pol between the tandem repeat PR domains resulted in diminished Gag cleavage efficiency.

## Conclusions

Our study indicates that the Gag cleavage enhancement effect incurred by over-expressed HIV-1 PR is reduced following the placement of p6pol between the tandem repeat PR domains. This supports the proposal that p6pol plays a negative role in the process of PR activation.
